# A deep learning based fusion of RGB camera information and magnetic localization information for endoscopic capsule robots

**DOI:** 10.1007/s41315-017-0039-1

**Published:** 2017-11-23

**Authors:** Mehmet Turan, Jahanzaib Shabbir, Helder Araujo, Ender Konukoglu, Metin Sitti

**Affiliations:** 1 Physical Intelligence Department, Max Planck Institute for Intelligent Systems, Stuttgart, Germany; 2 Institute for Systems and Robotics, Universidade de Coimbra, Coimbra, Portugal; 3 Computer Vision Laboratory, ETH Zurich, Zurich, Switzerland

**Keywords:** Deep Learning based Sensor Fusion, Endoscopic Capsule Robots, RNN-CNN (RNN:Recurrent Neural Network, CNN: Convolutional Neural Network)

## Abstract

A reliable, real time localization functionality is crutial for actively controlled capsule endoscopy robots, which are an emerging, minimally invasive diagnostic and therapeutic technology for the gastrointestinal (GI) tract. In this study, we extend the success of deep learning approaches from various research fields to the problem of sensor fusion for endoscopic capsule robots. We propose a multi-sensor fusion based localization approach which combines endoscopic camera information and magnetic sensor based localization information. The results performed on real pig stomach dataset show that our method achieves sub-millimeter precision for both translational and rotational movements.

## Introduction

Robot localization denotes the robot’s ability to establish its position and orientation within the frame of reference. Different sensors used in medical milliscale robot localization have their own particular strengths and weaknesses, which makes sensor data fusion an attractive solution. Monocular visual-magnetic odometry approaches, for example, have received considerable attention in mobile robotic sensor fusion literature. In general, localization techniques for endoscopic capsule robots can be categorized into three main groups: electromagnetic wave-based techniques; magnetic field strength-based techniques and hybrid techniquesUmay et al. (2017).

In recent years, numerous electromagnetic wave-based approaches like time of flight and difference of arrival (ToF and TDoA)-, received signal strength (RSS)-, RF identification (RFID)- and angle of arrival (AoA) based methods have been proposed Wang et al. (2011); Fischer et al. (2004); Wang et al. (2009); Ye (2013); Hou et al. (2009).

In magnetic localization systems, the magnetic source and magnetic sensor system are the essential components. The magnetic source can be designed in different ways: a permanent magnet, an embedded secondary coil, or a tri-axial magnetoresistive sensor. Magnetic sensors located outside the human body detect the magnetic flux density in order to estimate the location of the capsule (e.g., Popek et al. (2013), Natali et al. (2016), Yim and Sitti (2013)). One of the major advantages of utilizing magnetic field strength-based localization techniques is their successful coupling with magnetic locomotion systems. This could be achieved using magnetic steering, magnetic levitation, and remote magnetic manipulation. Other advantages include their robustness against attenuation by the human body. However, the disadvantage is that they experience interference from the environment. This could be handled by implementing additional hardware for handling the localization problem.

Another group of endoscopic capsule robot localization techniques is the hybrid techniques. These implement an integration of different sources at once such as RF sensors, magnetic sensors, and RGB sensors. The core idea is to integrate data from different sources which strengthen each other and can produce more accurate localization data. As a common approach Kalman filter and its derivatives are proposed to fuse RF electromagnetic signal data, magnetic sensor data, and video data. The first group of hybrid methods fuses RF and video signal Geng and Pahlavan (2016); Bao et al. (2015), whereas the second group focus on fusion of magnetic and RF signal data Umay and Fidan (2016); Geng and Pahlavan (2016); Umay and Fidan (2017) and the last group on fusion of magnetic and video data Gumprecht et al. (2013).

**Fig. 1:**
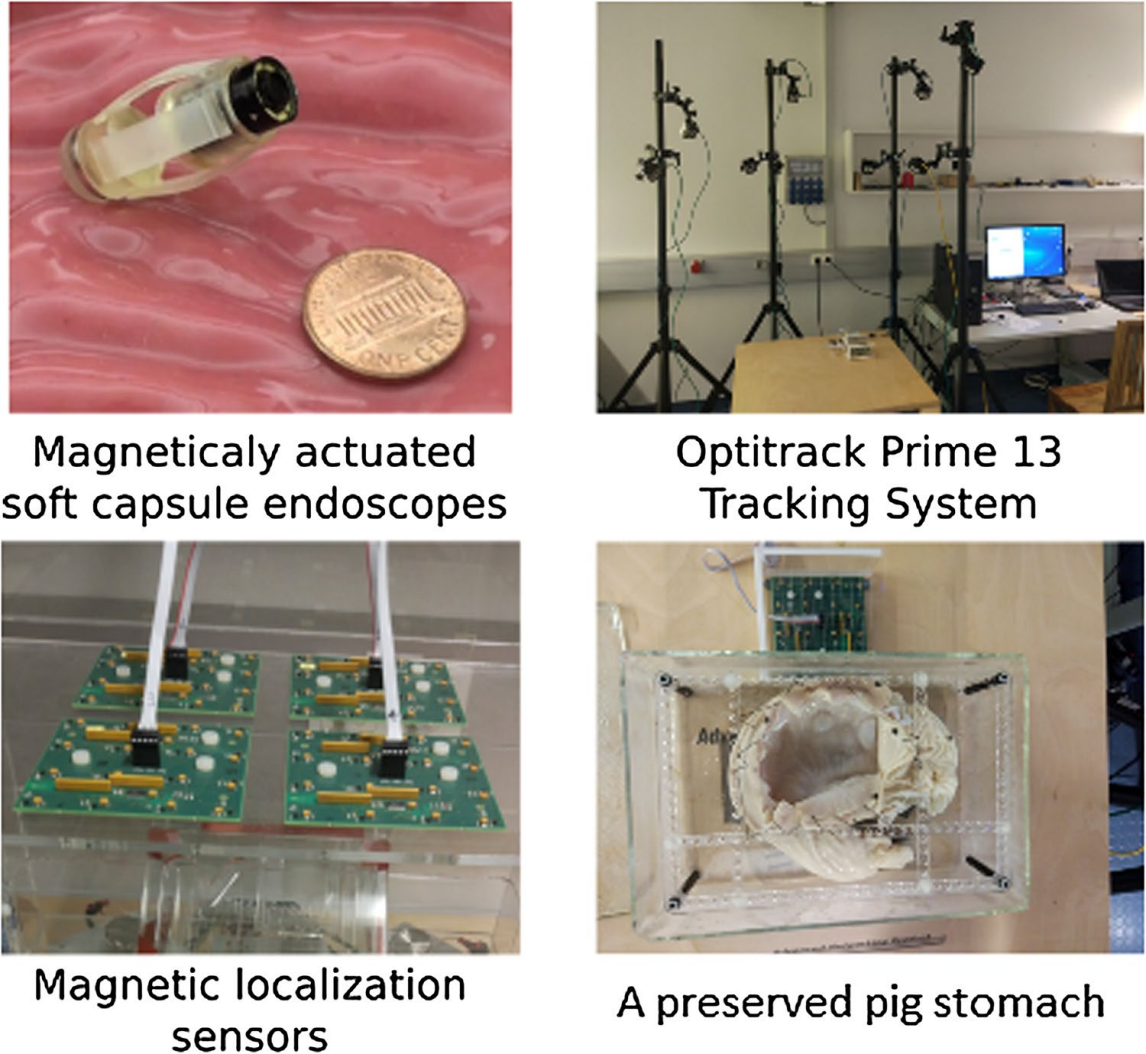
Experimental setup

Some other methods of localization utilize X-rays, MRI, computed tomography (CT), or ultrasound sensing Arshak and Adepoju (2006) and $\gamma$ rays Than et al. (2014). However, they all have their respective drawbacks of radiation hazards. MRI hardware is costly and presents (Fig. 1) additional design restrictions, and ultrasound sensing acquire planar pictures that might not intersect with the capsule robot. Inspired by the recent success of deep-learning models for processing raw, high-dimensional data, we propose in this paper a sequence-to-sequence deep sensor fusion approach for endoscopic capsule robot localization.

## System architecture details

Regardless of the algorithm, traditional monocular visual odometry solutions are subject to scale drift and ambiguity. It is proven that sophisticated loop closure methods can be helpful to reduce scale drift. However, scale ambiguity requires fusion of external information to be solved which not only resolves the scale ambiguity but also increases the pose estimation accuracy. With that motivation, we developed a deep neural network approach which fuses hand-eye calibrated and synchronized RGB camera information with magnetic localization information. Figure 3 shows the system architecture diagram of our sequence-to-sequence learning approach consisting of:

1. Optical Flow estimation.
2. CNN based feature vector extraction.
3. LSTMs based sensor fusion.

**Fig. 2:**
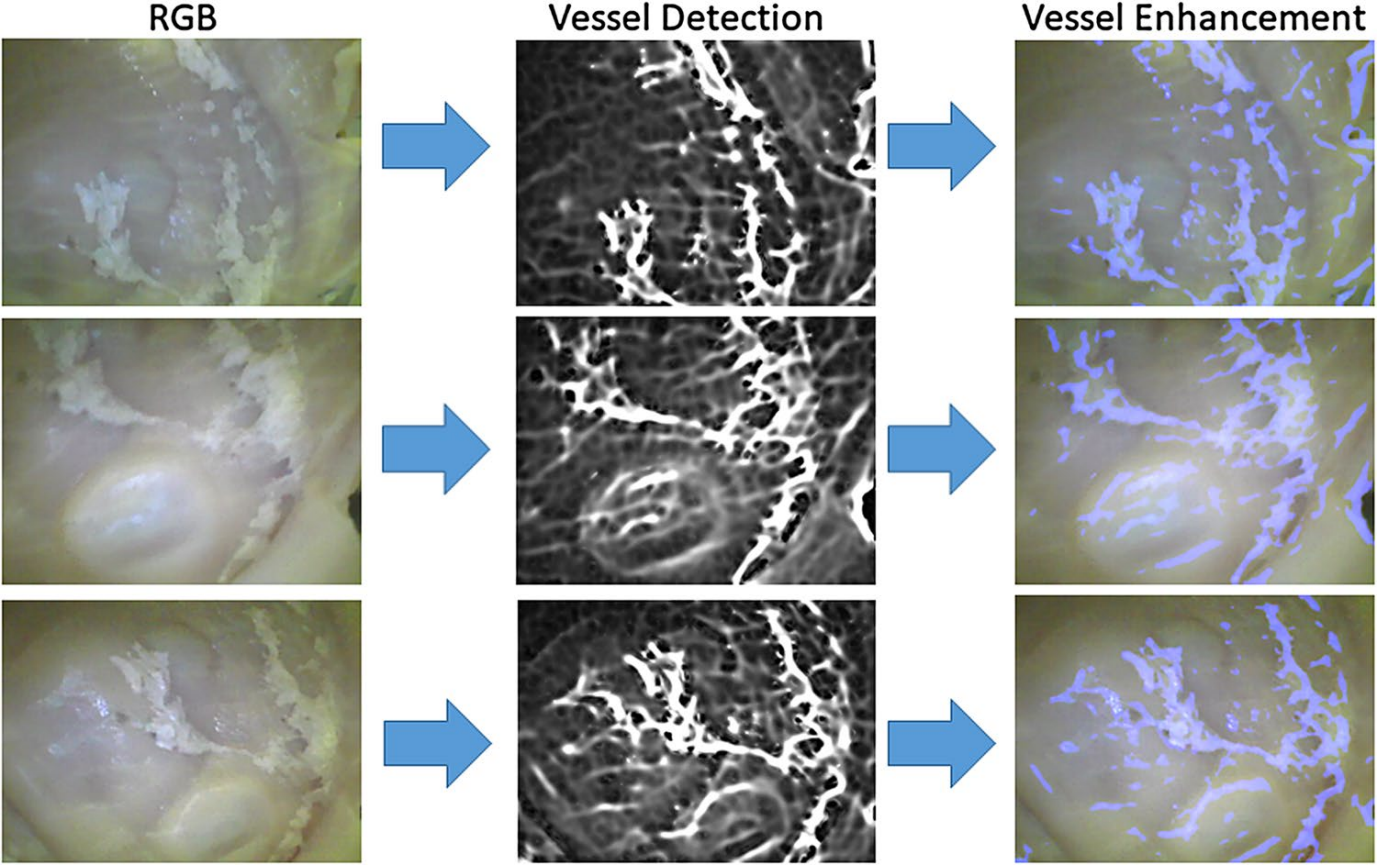
Vessel detection and enhancement

Before optical flow estimation, input frames are preprocessed by a vessel enhancement module which aims to emphasize unique features on the organ tissue (Fig. 2). As a next step, keyframe detection module examines each endoscopic camera frame and identify keyframes. Magnetic localization data coming from 2D Hall sensor array is a 6 dimensional vector containing the x, y, z position components and x-, y- and w orientation parameters in quaternion format (rotational z-degree is missing). The output of the network is a 7 dimensional vector consisting of x-, y-, z- translation and 4 orientation parameters in quaternion format.

### Preprocessing

Even though the beauty of deep learning is told to be its success and easiness to process raw input data without inquiring any pre-and post processing, we do preprocessing since it increases the accuracy of our method upon our observations we made during evaluations. This section explains the preprocessing operations we applied on the raw RGB image data before passing it into the deep neural network. The operations include vessel detection, enhancement and keyframe selection.

#### Multi-scale vessel enhancement

Since endoscopic images have mostly homogeneous and poorly textured areas, our framework starts with a vessel enhancement operation inspired from Frangi et al. (1998). Proposed approach enhances blood vessels by analyzing the multiscale second order local structure of an image. First, we extract the Hessian matrix:

1 $$\begin{aligned} H=\begin{bmatrix} I_{xx}&I_{xy}\\ I_{yx}&I_{yy} \end{bmatrix} \end{aligned}$$

where *I* is the input image, and $I_{xx}$, $I_{xy}$, $I_{yx}$, $I_{yy}$ the second order derivatives, respectively. Secondly, eigenvalues $\left| \lambda _{1} \right| \le \left| \lambda _{2} \right|$ and principal directions $u_{1}$, $u_{2}$ of the Hessian matrix are extracted. The eigenvalues and principal directions are then ordered and analyzed to decide whether the region belongs to a vessel. To identify vessels in different scales and sizes, multiple scales are created by convolving the input image and the final output is taken as the maximum of the vessel filtered image across all scales. For further details of the mathematical equations, the reader is referred to the original paper of [?]. Figure 2 shows input RGB images, vessel detection and vessel enhancement results for different frames.

#### Keyframe selection

Due to the incremental and slow motion of the capsule robot inside the inner organ, endoscopic videos generally contain numerous frames with similar and redundant content. Thus, an algorithm has to be developed to skip frames with similar overlapping features. This procedure is called keyframe selection. We developed a method based on optical flow interpretation between consecutive frame pairs. The output of optical flow algorithm is the vector values of each pixel which we sum the magnitudes of and normalize by dividing it with the total number of pixels. If the normalized value exceeds a pre-defined threshold value of 20 pixels, the overlap between the corresponding frames is less than $75\%$ and it is identified as a keyframe. Conversely, if it does not exceed the threshold then there is a high overlap between the frame pair. This procedure is summarized below:

1. Choose a candidate keyframe and extract Farneback optical flow between this and the reference keyframe.
2. Compute the magnitude of the extracted optical flow vector for each pixel.
3. Calculate the cumulative value by summing up all the magnitude values.
4. Normalize the cumulative value by the total number of pixels.
5. If the normalized cumulative value is less than $\tau$ then go to the next frame. Otherwise, identify the candidate key frame as a key frame and repeat the process.

### Optical flow extraction

**Fig. 3:**
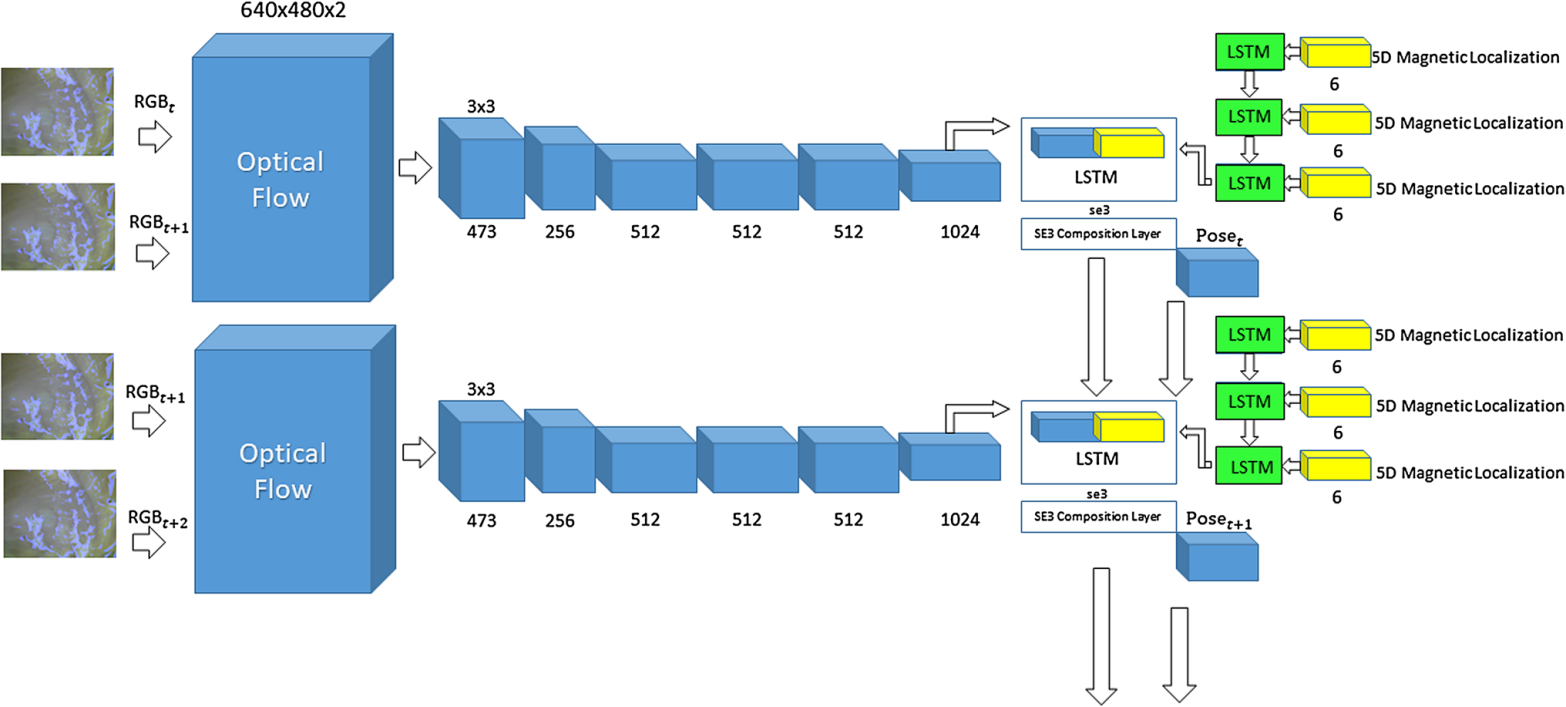
System architecture diagram

**Fig. 4:**
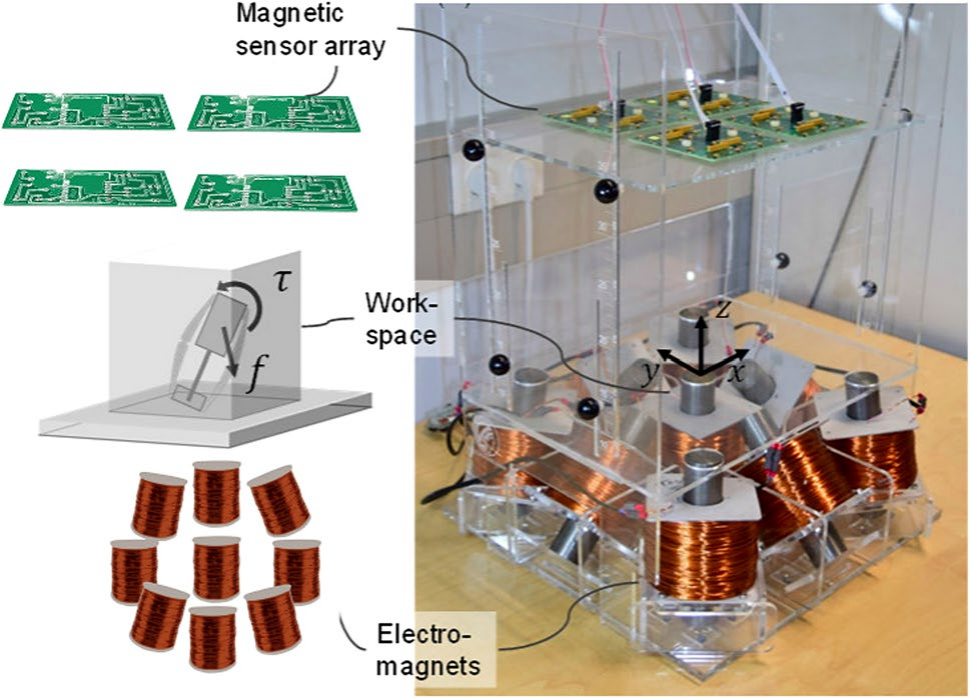
Magnetic localization system

In deep learning based applications, there is in general a tendency to serve raw input images into the neural network without any preprocessing so that neural network can decide by itself how to organize the raw information. Contrary to that, we do not use raw images, instead we extract optical flow from consecutive keyframes. This way, we want to force CNN to focus more directly on the motion dynamics between frames rather than redundant unnecessary information. To achieve real time performance, we make use of GPU for optical flow estimation. Several optical flow algorithms such as Lucas-Kanade method (Cornelius and Kanade 1984), Buxton-Buxton method (Beauchemin and Barron 1995) and the Black Jepson method were tested. The Farneback optical flow estimation based on polynomial expansion (Farnebäck 2003) out-performed all other optical flow methods by having the lowest reprojection error (Fig. 4). The input information of the CNN needs to be discrete which arises the need to quantify the optical flow vector. The resolution of the input image is 640 $\times$ 480, so the maximum value of the quantification is devised to be the diagonal length of the image resolution, and minimum quantification value is set to zero. The quantification range is divided into 1024 intervals and the resulting quantized x and y values were concatenated for each pixel (Fig. 3).

### Magnetic localization system

As described in Fig. 4, our magnetic localization system consists of a magnetic Hall sensor array for localization and electromagnets for actuation of the endoscopic capsule robot. Our magnetic localization technique is able to measure 5-DoF absolute pose of the untethered meso-scale magnetic robot in real-time. A Hall-effect sensor array measures magnetic field at several locations from the capsule robot, whereas a computer-controlled electromagnetic coil array provides actuator’s magnetic field. The core idea of our localization technique is separation of capsule’s magnetic field from actuator’s known magnetic field, which is realized by subtracting actuator’s magnetic field component from the acquired magnetic data. Finally, noise effects are reduced by second-order directional differentiation. For curious readers, further details of our magnetic localization technique can be found in Son et al. (2016).

### Deep CNN-RNN architecture for sensor fusion

We propose a sensor fusion architecture consisting of CNN layers for feature extraction from quantized optical flow vector, multi-rate long short-term memories (LSTMs) for frequency adjustment, and a core LSTM unit for fusion of flattened feature vector and magnetic localization information. The details of the system architecture can be seen in Fig. 3. The core part of the architecture is inspired and modified from Clark et al. (2017). For the implementation, Keras library with Theano back-end was used which provides a modifiable framework, enables multi-GPU training which accelerates the computational procedure. The learning rate was initialized to 0.001 reducing as the epochs of the training continues. Adaptive moment estimation (Adam) method was used to optimize the goal function. We trained our algorithm on an Amazon EC2 p2.xlarge GPU compute instance. The list of the parameters is as follow:

- learning rate: 0.001
- momentum1: 0.9
- momentum2: 0.999
- epsilon: $$10^{-8}$$
- solver type: Adam
- batch size: 64
- GPU: NVIDIA K80

**Fig. 5:**
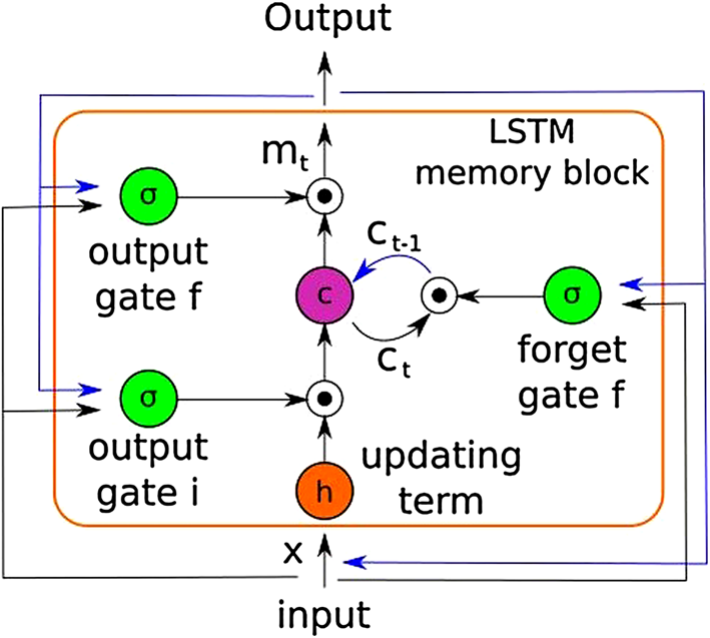
Information flow through the hidden units of the LSTM

With using LSTM, we pursue to learn the complex motion dynamics of endoscopic capsule robot and try to let the neural network describe sequential dependencies across frames which require extensive engineering in case of manual modeling. Contrary to the traditional LSTM, we connect the output pose of the current core LSTM as input to the core LSTM of the next timestep so that odometry can benefit from the information of past frames thanks to its hidden memory lasting over time. With the help of this hidden memory, LSTM can encode the previously gained knowledge up to time step *t* and use it for posterior estimations. An exponential map in the *SE*(3) composition layer is used to convert *se*(3) data to the special euclidean group *SE*(3) Clark et al. (2017). In our architecture, each LSTM layer has 200 hidden states. To regress the 6-DoF pose, we trained the architecture on the following objective loss function:

2 $$\begin{aligned} loss(I) = \Vert \hat{\mathbf {x}} - \mathbf {x}\Vert _2 + \beta \Vert \hat{\mathbf {q}} - \mathbf {q}\Vert _2 \end{aligned}$$

where $\mathbf {x}$ is the translation vector and $\mathbf {q}$ is the quaternion vector for a rotation. A balance $\beta$ must be kept between the orientation and translation loss values which are highly coupled as they are learned from the same model weights. Experimental results show that the optimal $\beta$ is given by the ratio between expected error of position and orientation at the end of training session (Fig. 5). The back-propagation algorithm is used to determine the gradients of the network weights which are passed into the Adam optimization. The moments of the gradient are calculated using exponential moving average in addition to exponentially decaying average of past gradients, which also corrects the bias.

## Dataset

**Fig. 6:**
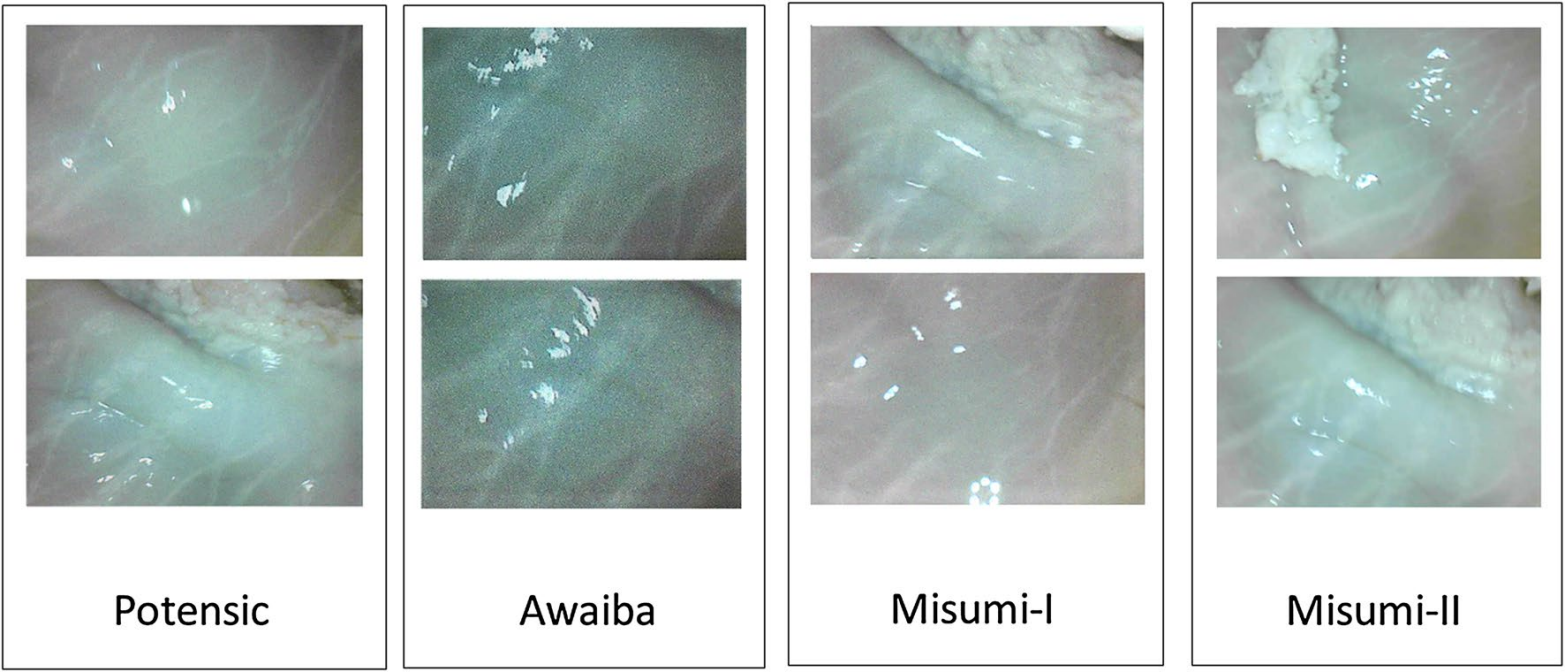
Sample images from dataset

This section introduces the experimental setup and explains how the training and testing datasets were created. The dataset was recorded on five different real pig stomachs (see Fig.1). In order to ensure that our algorithm is not tuned to a specific camera model, four different commercial endoscopic cameras were employed. For each pig stomach and camera combination, 3000 frames were acquired, which makes 60,000 frames for four cameras and five pig stomachs in total. 40,000 frames were used for training, whereas the remaining 20,000 frames were used for evaluation. Sample real pig stomach frames are shown in Fig. 6 for visual reference. During video recording, an Optitrack motion tracking system consisting of eight Prime-13 cameras was utilized to obtain 6-DoF localization ground-truth-data with sub-millimeter accuracy (see Fig. 1).

## Evaluation

We evaluate the performance of our system both quantitatively and qualitatively in terms of trajectory estimation. We also report the computational time requirements of the method.

### Trajectory estimation

The absolute trajectory (ATE) root-mean-square error metric (RMSE) is used for quantitative comparisons, which measures the root-mean-square of Euclidean distances between all estimated endoscopic capsule robot poses and the ground truth poses. We created six different trajectories with various complexity levels. Overfitting, which would make the resulting pose estimator inapplicable in other scenarios, was prevented using dropout and early stopping techniques. The dropout regularization technique, which samples a part of the whole network and updates its parameters based on the input data, is an extremely effective and simple method to avoid overfitting.

**Fig. 7:**
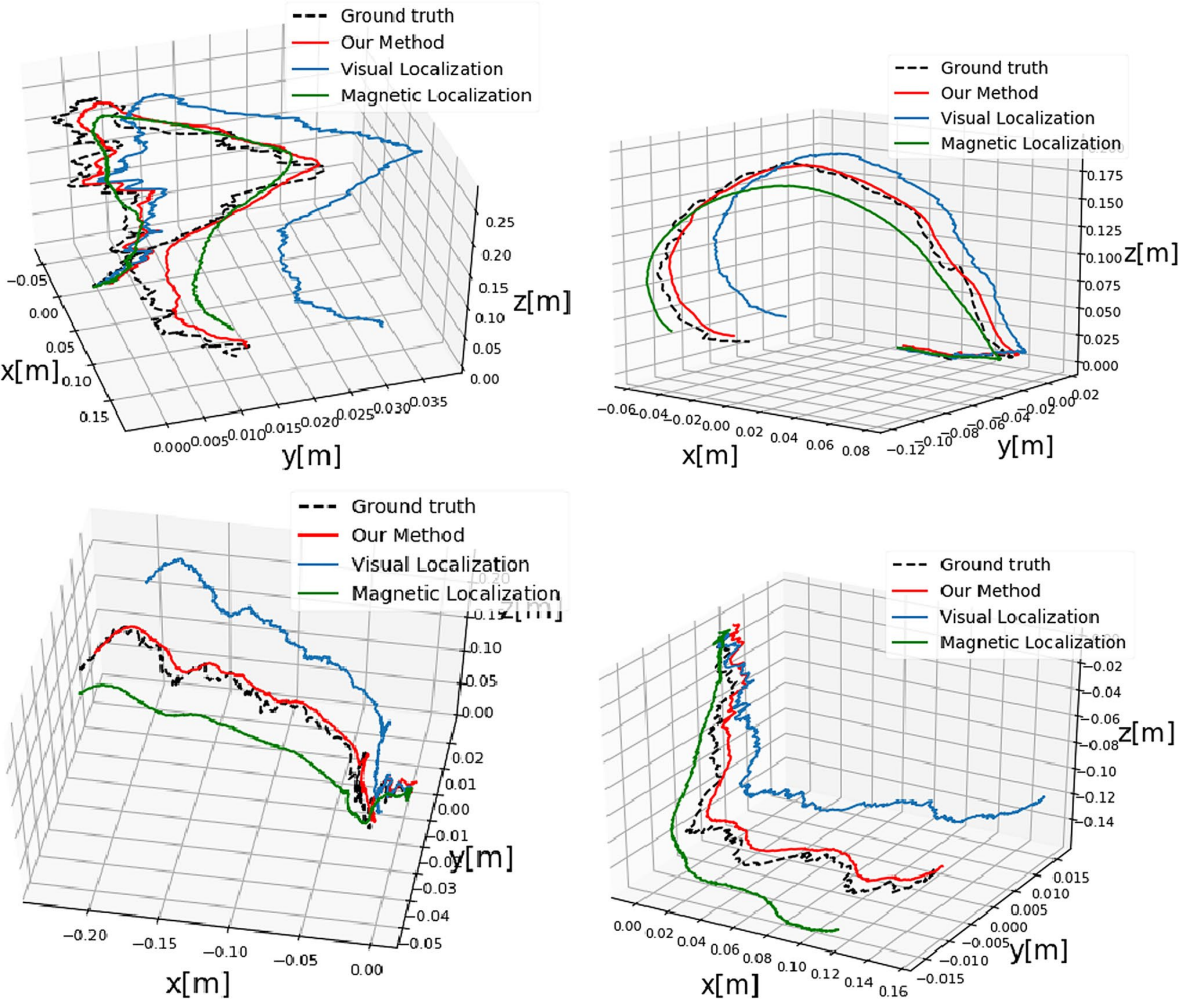
Plotted trajectories

**Fig. 8:**
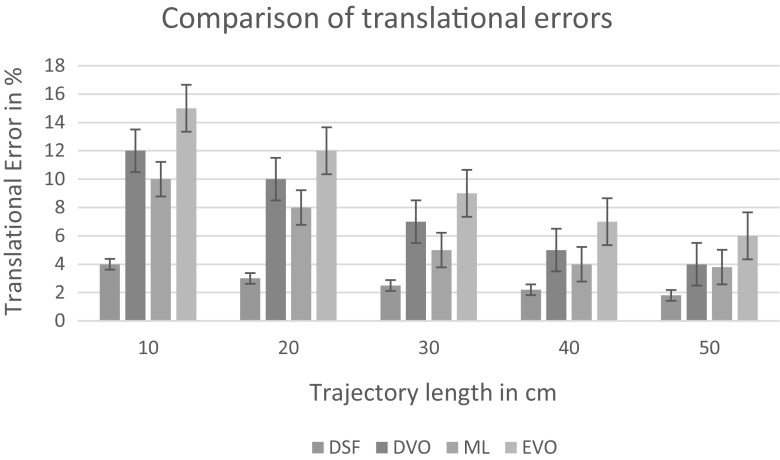
Trajectory length versus translation error

**Fig. 9:**
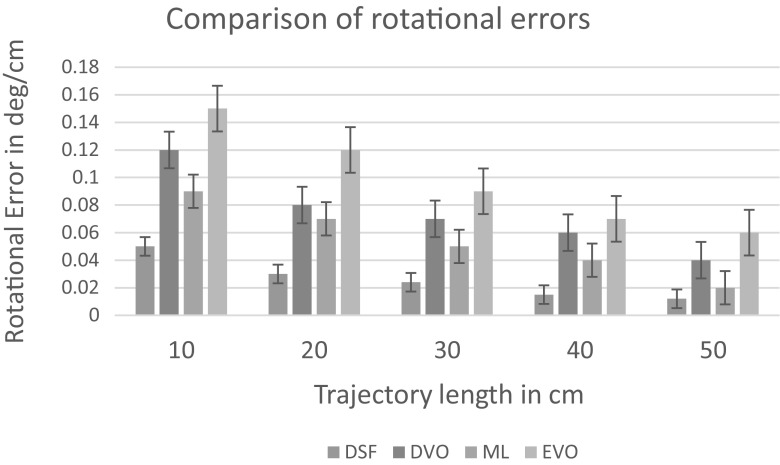
Trajectory length versus rotation error

Early stopping is another widely used technique to prevent overfitting of a complex neural network architecture optimized by a gradient-based method. We strictly avoided the use of any image frames from the training session for the testing session. We compared the performance of our deep fusion approach with the odometry approach proposed in Turan et al. (2017) which we call endoscopic visual odometry (EVO) , magnetic localization approach proposed in Son et al. (2017) and with the same CNN-RNN system in Fig. 3 except we disabled the magnetic localization fusion path. We call this last configuration deep visual odometry (DVO). The average translational and rotational RMSEs for deep sensor fusion, EVO, magnetic localization and DVO against different path lengths are shown in Figs. 8 and 9, respectively.The results indicate that deep sensor fusion clearly outperforms all other configurations, while magnetic localization outperforms EVO and DVO. Some qualitative tracking results and corresponding ground truth trajectories for deep fusion approach, DVO and magnetic localization are demonstrated in Fig. 7 for visual reference. As seen in sample trajectories, deep fusion is able to stay close to the ground-truth pose values for even complex, fast rotational and translational motions, where both EVO and magnetic localization by themselves clearly deviate from the ground-truth trajectory. Thus, we can conclude that deep fusion makes effective use of both sensor data streams Based on our evaluations, we presume that the hybrid use of the CNN-LSTM architecture enabled learning from both magnetic and visual information effectively which led to optimal localization results. We run our training and testing sessions both on an Amazon EC2 p2.xlarge machine instance. The duration of the training session was 8 h and 37 min. The 6-DoF pose estimation per image pair and magnetic data took 35 ms Figs. 8 and 9.

## Conclusion

In this study, we presented, to the best of our knowledge, the first sensor fusion method based on deep learning for endoscopic capsule robots. The proposed CNN-RNN architecture based fusion approach is able to achieve simultaneous learning and sequential modeling of motion dynamics across frames and magnetic data streams. Since it is trained in an end-to-end manner, there is no need to carefully hand-tune the parameters of the system except the hyperparameters. In the future, we will incorporate controlled actuation into the scenario to investigate a more complete system, and additionally we will seek ways to make the system more robust against representational singularities in the rotation data.
